# Mucormycosis: Battle with the Deadly Enemy over a Five-Year Period in India

**DOI:** 10.3390/jof4020046

**Published:** 2018-04-06

**Authors:** Jagdish Chander, Mandeep Kaur, Nidhi Singla, R. P. S. Punia, Surinder K. Singhal, Ashok K. Attri, Ana Alastruey-Izquierdo, Alberto M. Stchigel, Jose F. Cano-Lira, Josep Guarro

**Affiliations:** 1 Departments of Microbiology, Government Medical College Hospital, Sector 32-B, Chandigarh, PIN 160030, India; jchander@hotmail.com (J.C.); drmandeepkaur@gmail.com (M.K.); 2 Pathology, Government Medical College Hospital, Sector 32-B, Chandigarh, PIN 160030, India; drpunia@gmail.com; 3 Otorhinolaryngology, Government Medical College Hospital, Sector 32-B, Chandigarh, PIN 160030, India; singhalsks@gmail.com; 4 General Surgery, Government Medical College Hospital, Sector 32-B, Chandigarh, PIN 160030, India; attriak@yahoo.com; 5 Mycology Reference Laboratory, Spanish National Center for Microbiology, Instituto de Salud Carlos III, 28029 Madrid, Spain; anaalastruey@isciii.es; 6 Mycology Unit, Medical School and IISPV, Universitat Rovira I Virgili, 43201 Reus, Spain; albertomiguel.stchigel@urv.cat (A.M.S.); jose.cano@urv.cat (J.F.C.-L.); josep.guarro@urv.cat (J.G.)

**Keywords:** Mucormycosis, *Rhizopus*, *Saksenaea*, *Apophysomyces*, *Lichtheimia*

## Abstract

Mucormycosis is an emerging opportunistic fungal infection. Increasing immunocompromization, widespread use of antibacterial and antifungal agents (such as voriconazole prophylaxis), carcinomas, transplantation and lifestyle diseases such as diabetes are the main contributors to this situation. The predominant clinical manifestations of mucormycosis vary from host to host, with rhino-orbital-cerebral, pulmonary, cutaneous, and gastrointestinal infections being the most common. In India, the prevalence of mucormycosis is approximately 0.14 cases/1000 population, which is about 70 times the worldwide-estimated rate for mucormycosis. The present study was undertaken over a period of five years (January 2009–December 2014) to determine the prevalence of mucormycosis. The samples suspected of mucormycosis were examined by direct KOH wet mount and cultured on Sabouraud’s dextrose agar without actidione and on blood agar as per standard mycological techniques. Histopathological correlation was done for most of the cases. Antifungal susceptibility testing was performed by the EUCAST reference method. We identified a total of 82 cases of mucormycosis out of a total of 6365 samples received for mycological culture and examination during the said time period. Out of these, 56 were male patients and 27 were females. Most common presentation was rhino-orbito-cerebral (37), followed by cutaneous (25), pulmonary (14), oral cavity involvement (4) and gastrointestinal (2). The most common risk factors were diabetes and intramuscular injections. The fungi isolated were *Rhizopus arrhizus* (17), *Apophysomyces variabilis* (12), *R. microsporus* (9), *Lichtheimia ramosa* (8), *Saksenaea erythrospora* (5), *Syncephalastrum racemosus* (4), *R. homothallicus* (2), *Rhizomucor pusillus* (1), *Mucor irregularis* (1) and *A. elegans* (1). The mainstay of the treatment was amphotericin B, along with extensive surgical debridement whenever feasible. Most of the patients (50) recovered, but 25 died. The rest of the patients left against medical advice. “Nip in the Bud” should be the mantra for clinicians/surgeons for a favorable prognosis. Early diagnosis, prompt institution of appropriate antifungal therapy, surgical debridement whenever necessary, knowledge of risk factors and their timely reversal is the key for management.

## 1. Introduction

Mucorales are ubiquitous fungi and are commonly found in decaying organic matter. They have been isolated in the laboratories as contaminants for a long time. However, changing host environments are causing their emergence as potential pathogenic organisms leading to high morbidity and very high and quick mortality. The disease caused by them has been reported all over the world and although associated with immunocompromization states like carcinomas or immunosuppressive therapy, it also affects a newer range of susceptible hosts like diabetics, neutropenics, patients on desferroxamine therapy, etc. [1]. Chakrabarti et al. have estimated a prevalence of 0.14 per 1000 cases of diabetics in India, which is about 80 times the prevalence of mucormycosis in developed countries. If we consider that currently the number of diabetics in India is nearly 62 million and they are likely to cross the 100 million mark by 2030, the burden of mucormycosis in India in future can be well imagined [2].

Mucormycosis is a disease difficult to diagnose in clinical and laboratory settings. A high index of suspicion on the clinician side with the need to send an appropriate sample to the laboratory is a prerequisite. It is not easy to isolate and maintain in the laboratory since their poorly septate hyphae can lose the vital cytoplasm at the least manipulation. Moreover, the awareness among microbiologists is still low, with most isolates classified as “*Mucor* or *Rhizopus*” without further identification. Newer species are recognized (*Apophysomyces, Mucor irregularis*), new areas of isolation have been identified (isolated renal mucormycosis), and mucormycosis has broadened its host range (involving both immunocompetent and immunocompromised). Risk factors are different (being more common in diabetics, neutropenics than AIDS patients), and it is difficult to treat (not responding to the most common antifungal drugs used, i.e., azoles with posaconazole and isavuconazole being the exception). The disease warrants the efficient training of clinicians and surgeons in dealing with this fungus which is angioinvasive and often the hyphae have invaded deep into the healthy tissue before debridement is done [3]. A wide area of healthy tissue needs to be debrided to get rid of the fungus from the surroundings of the wound.

Presently, Phylum Glomeromycota is divided into four subphyla: Mucoromycotina, Entomophthoromycotina, Kickxellales and Zoopagomycotina (elevating the orders Mucorales and Entomophthorales to a subphylum status). The mucormycetes (previously Zygomycetes) belong to the order Mucorales and involve 6 main families Syncephalastraceae (Genus *Syncephalastrum*), Saksenaeaceae (genera *Saksenaea* and *Apophysomyces*) Cunninghamellaceae (genus *Cunninghamella*), Mucoraceae (genera *Mucor*, *Rhizopus*, *Rhizomucor* and *Actinomucor*), Thamnidiaceae (*Cokeromyces*) and Lichtheimiaceae (genus *Lichtheimia*) [4]. About 70% to 80% of cases of mucormycosis are caused by fungi belonging to the genera *Rhizopus*, *Mucor* and *Lichtheimia*. The disease is commonly acquired by inhalation of sporangiospores. Other routes include ingestion of spores, direct implantation into injured skin, and trauma with contaminated soil, intravenous or intramuscular injections. The most common manifestation of the disease is rhino-orbito-cerebral (ROC) mucormycosis, mostly seen in diabetics [5].

## 2. Material and Methods

The present prospective observational study was conducted in the Department of Microbiology, Government Medical College Hospital, Chandigarh, in association with the Department of Pathology and other clinical Departments from which samples were received to find out the prevalence of mucormycosis in our region. Chandigarh is located in foothills of the Shivalik range of the Himalayas in northwest India. The study was carried out over a period of five years from January 2010 to December 2014. All patients suspected of suffering from mucormycosis in our hospital were examined for the presence of fungi. Depending upon the site of infection, the samples (tissues, biopsies, fluids, etc.) were received in the microbiology laboratory and were processed using standard mycological techniques [6]. All samples were initially examined microscopically by performing potassium hydroxide (KOH) wet mounts followed by putting culture on two tubes of Sabouraud dextrose agar (SDA) and kept at 25 °C and 37 °C. Histopathological correlation was also done. The macro and micro characteristics of the fungal growth were examined on Lactophenol cotton blue (LCB). Sporulation was tried on potato dextrose agar and oat meal agar. The final identification of isolates was performed by molecular sequencing at Mycology Reference Laboratory, Spanish National Center for Microbiology, Instituto de Salud Carlos III, Madrid (Spain) and Mycology Unit, Medical School and IISPV, Universitat Rovira I Virgili, Reus, Spain. Molecular sequencing was done from the fungal growth, the amplification and nucleotide sequencing of the internal transcribed spacer (ITS) region and the D1–D3 domains of the 28S nuclear ribosomal RNA (rRNA) gene (larger subunit (LSU)) were carried out with primer pair ITS5/LR5, and the obtained sequences were compared with those of the ex-type strains (reference strains). The antifungal susceptibility testing of the isolates was performed for amphotericin B, itraconazole, voriconazole, posaconazole, ravuconazole, caspofungin, micafungin, anidulafungin and terbinafine using EUCAST microdilution method [7]. Growth was examined visually after incubation at 35 °C for 48 h and the MICs or MECs were determined. *Aspergillus fumigatus* ATCC 2004305 and *Aspergillus flavus* ATCC 2004304 were used as quality control strains.

## 3. Results

Over a study period of five years, a total of 82 patients were diagnosed to be suffering from mucormycosis based on microbiological and/or histopathological examination (HPE) of the clinical samples. The profile of the patients is given in Table 1.

Of the total patients, 55 were male and 27 were female. Most of them were adults, except 4, who were less than 18 years (2 were < 10 years and two were 12 and 15 years). The most commonly affected age group was that between 31–60 years. A large number of patients (*n* = 51) presented with diabetes mellitus. Rhino-orbito-cerebral mucormycosis was the most common presentation followed by cutaneous infection. Sixty isolates were grown on culture, while in the others, the diagnosis was made either on direct KOH wet mount or on the histopathological examination. The antifungal treatment was started in around 82.9% (*n* = 68) of the cases, 78% (*n* = 64) of them were managed with amphotericin B (either conventional or liposomal formulation). Thirty-one of the patients died while the outcome of 8 was unknown, as they left the hospital against medical advice and could not be followed up. The species isolated are given in Table 2.

*Rhizopus arrhizus* was the most common, followed by *Apophysomyces variabilis*. The antifungal susceptibility was obtained in 40 isolates (Table 3). Failure to sporulate was the major hindrance in most of the isolates. The MIC ranges for amphotericin B and posaconazole were 0.03- >16 mg/L and 0.06- >8 mg/L, respectively. A high degree of resistance was observed in *Apophysomyces elegans* and *Rhizopus homothallicus*, in which all the antifungals exhibited high MICs.

## 4. Discussion

*Rhizopus* was the genus most commonly isolated from the patients and most easily recognized in our study, *R. arrhizus* being the species most common, although other species of the genus, such as *R. microsporus* and *R. homothallicus*, were also found. *R. arrhizus* has two varieties: var. *arrhizus* and var. *delemar*. Both varieties have a worldwide distribution and have no difference in ecology and epidemiology; however, as per multilocus studies and AFLP, they have been recognized as two different phylogenetic species [8]. Members of the *R. microsporus* group have been separated into several varieties based upon different morphologic characteristics: *R. microsporus* var. *microsporus*, *R. microsporus* var. *rhizopodiformis*, *R. microsporus* var. *chinensis*, *R. microsporus* var. *azygosporus*, *R. microsporus* var. *oligosporus* and *R. microsporus* var. *tuberosus* [9]. However, recently it has been proposed that they be reported as *R. microsporus* only, without mentioning variety, as they are all identical by sequence analysis. Previously, there have been reports of *R. microsporus* causing lung infections, cellulitis in the leg of a diabetic, and nosocomial infection in preterm infants via wooden tongue depressors [10]. Here, we have isolated 9 *R. microsporus* (5 pulmonary samples, 2 hard palate tissue and 2 rhino orbital). Another species isolated was *R. homothallicus* in two patients. The species can be easily identified by its golden brown zygospores and unequal suspensor cells. Previously, Chakrabarti et al. had also reported its isolation from cases of cavitary pulmonary lesions [11]. In our study, one patient suffered a rhino-orbito-cerebral infection and other showed features of pulmonary mucormycosis with the fungus isolated from a BAL. In a recent study, such species caused rhinocerebral and cutaneous mucormycosis [12].

The second-most common genus isolated was *Apophysomyces* (12 *A. variabilis* 1 *A*. *elegans*)*. A. elegans* was first isolated by Misra et al. in 1979 from soil samples collected from a mango orchard in northern India and was the only species in the genus till recently [13]. Now, five species of *Apophysomyces* are known, i.e., *A. elegans*, *A. variabilis*, *A. ossiformis*, *A. trapeziformis*, and a newly recognized species *A. mexicanus* [14]. Although infections like ROC or renal have been reported previously, the species of this genus are mainly associated to necrotizing fasciitis. *A. trapeziformis* was identified in cases of necrotizing soft tissue infections in victims of a tornado in Joplin, Missouri [15]. *A. elegans* and *A. variabilis* have been frequently reported by our institution in the past. The majority of our isolates were severe necrotizing fasciitis by *A. variabilis*. Many cutaneous cases were immunocompetent with major a risk factor being I/M injections given in gluteal area. Most of the time, patients belonged to rural areas, were illiterate, had been treated by quacks, and the injection had been given through clothes without open skin disinfection [16].

*Lichtheimia* was isolated in 8 patients (7 *L. ramosa* and 1 *L. corymbifera)*. *Lichtheimia*, renamed from genus *Absidia*, includes four species: *L. corymbifera*, *L. ramosa*, *L. blakesleeana* and *L. hyalospora*, although only the two first have been reported to cause human infections. The role of *L. ramosa* in causing human infections is increasingly recognized; indeed, it is considered that a significant number of previously reported *L. corymbifera* infections may have actually been *L. ramosa* [17]. In the microbiology laboratory, a fungus of this group with abundant circinate side branches on the sporangiophores and pleomorphic giant cells with finger-like projections can give sufficient clues to suspect *Lichtheimia* as a possible isolate.

*S. erythrospora* is another significant finding in this study. The genus *Saksenaea* includes three species, i.e., *S. vasiformis*, *S. erythrospora* and *S. oblongispora* [18]. These fungi are known to cause necrotizing cutaneous infections just like *Apophysomyces*. *Saksenaea* is known to be associated with soft tissue infections in healthy and immunocompetent persons. Previously, we had reported *S. vasiformis* from our institution; however, this time, we identified 5 cases of primary cutaneous infections by *S. erythrospora* in immunocompetent individuals after traumatic implantation. In four patients, intramuscular injection into the gluteal region was the main predisposing factor while upper limb involvement, following medicated adhesive tape application, was seen in one patient. The best of our knowledge, this is the first time that *S. erythrospora* has been reported from India [19]. In the literature, *S. erythrospora* has been reported to cause infections in patients of combat injuries [20].

We isolated 4 strains of *Syncephalastrum*, 2 were with pulmonary signs and symptoms, one with nasal infection and the fourth patient had vesicular lesions all over the body (disseminated *Varicella* with necrotizing fasciitis). The members of this genus have usually been isolated from onychomycosis cases. In the literature, fewer than 10 cases of infections by *Syncephalastrum* have been reported. Recently, a case of subcutaneous mucormycosis due to *Syncephalastrum* was reported [21].

Another significant isolation was of *M. irregularis* (previously *Rhizomucor variabilis*). The infection is known to have caused chronic infections in a Chinese province, in addition to two additional cases from southern India [22]. Outside Asia, cases have been reported in Japan, Australia, and USA [23]. We isolated it for the first time in North India in a farmer with lesions in the lower limbs [23].

In our study, most of the patients, 33 (40%), belonged to the age group within the range of 46–60 years, followed by 28 (34%) in the age group 31–45 years. Males were more commonly affected 67% (55/82). Previously, Roden et al. [24] had also reported the mean age of mucormycosis patients to be 38.8 years, with median age 40.0 years and a total of 65% of the infections in males.

Most of the patients were treated with antifungal drugs—82.9% (*n* = 68) cases—among which 78% (*n* = 64) received amphotericin B (either conventional or liposomal formulation). Most of the patients (50) recovered, but 25 died. The rest of the patients left against medical advice. There are various reasons for bad prognosis in mucormycosis; these mainly include delayed diagnosis, need for prolonged antifungal therapy along with surgical intervention, and thereby the need for extensive debridement. The infections are usually severe and have a very rapid course of spread and are often quite extensive by the time the patient reports to the clinician. Mucormycosis is generally an infection with bad prognosis; therefore, there is a need to remain vigilant regarding host groups, risk factors, epidemiology and early intervention for favorable outcome.

## Figures and Tables

**Table 1:** The distribution of patients and the risk factors of mucormycosis in a five-year study period.

	2010	2011	2012	2013	2014	Total
Number of cases reported	28	14	9	17	14	82
Male/Female	22/6	7/7	7/2	12/5	7/7	55/27
Age						
<15 years	1	1	-	-	1	3
15–30 years	4	3	1	2	-	10
31–45 years	11	4	2	7	4	28
46–60 years	11	4	4	7	7	33
>60 years	1	2	2	1	2	8
Risk Factors						
Diabetes mellitus	15	9	8	11	8	51
Intramuscular injection	4	2	-	3	3	12
Iron therapy/dialysis	2	-	-	-	-	2
Others (malignancy, underlying infection, post- surgery, trauma, etc.)	3	2	1	-	2	8
None	8	3	-	4	1	16
Site of mucormycosis						
Rhino-orbito-cerebral	14	9	6	6	2	37
Cutaneous	8	4	-	6	7	25
Pulmonary	1	1	3	4	5	14
Oral cavity	3	-	-	1	-	4
Gastrointestinal	2	-	-	-	-	2

**Table 2:** The year-wise distribution of Mucormycetes isolates in culture-positive cases of mucormycosis.

Number of Cases by Mucormycetes	2010	2011	2012	2013	2014	Total
*Apophysomyces elegans*	-	1	-	-	-	1
*Apophysomyces variabilis*	5	2	-	4	1	12
*Lichtheimia corymbifera*	-	-	1	-	-	1
*Lichtheimia ramosa*	5	-	-	2	-	7
*Mucor irregularis*	-	-	-	1	-	1
*Rhizopus arrhizus*	4	4	5	2	2	17
*Rhizopus microsporus*	1	1	1	3	3	9
*Rhizopus homothallicus*	1	-	-	1	-	2
*Rhizomucor pusillus*	-	1	-	-	-	1
*Saksenaea erythrospora*	-	-	-	1	4	5
*Syncephalastrum racemosum*	-	-	-	2	2	4
						60

**Table 3:** Antifungal susceptibility testing results of Mucormycetes.

Antifungal Tested	MIC/MEC (mg/L)	Mucormycetes Species (Number of Strains Tested)								
		*A. elegans* (1)	*A. variabilis* (10)	*L. ramosa* (5)	*M. irregularis* (1)	*R. arrhizus* (11)	*R. microspores* (7)	*R. homothallicus* (1)	*R. pusillus* (1)	*S. racemosum* (3)
AMB	MIC/MIC range	8	1–16	0.03–4	0.5	0.12–16	0.5–16	>16	0.3	0.06- >8
	MIC _50_	-	4	0.5	-	0.25	1	-	-	0.5
	MIC _90_	-	8	4	-	1	16	-	-	>8
ITC	MIC/MIC range	>8	0.12- >8	1–16	0.5	8–16	>8- >8	>8	0.5	0.06- >8
	MIC _50_	-	4	1	-	>8	>8	-	-	8
	MIC _90_	-	8	16	-	>8	>8	-	-	>8
VOR	MIC/MIC range	>8	8–16	8–16	>8	8–16	>8- >8	>8	>8	0.06- >8
	MIC _50_	-	>8	8	-	>8	>8	-	-	>8
	MIC _90_	-	>8	16	-	>8	>8	-	-	>8
RVC	MIC/MIC range	>8	0.5–8	2–4	-	0.25–8	-	-	1	-
	MIC _50_	-	0.5	4	-	2	-	-	-	-
	MIC _90_	-	4	4	-	8	-	-	-	-
PSC	MIC/MIC range	>8	0.12–8	0.25–0.5	1	0.25–8	2- >8	8	0.25	0.06->8
	MIC _50_	-	0.5	0.25	-	4	>8	-	-	>8
	MIC _90_	-	4	0.5	-	8	>8	-	-	>8
TBF	MIC/MIC range	8	0.12–8	1–32	>16	2–32	2- >16	>16	0.5	0.12->16
	MIC _50_	-	0.5	8	-	>16	>16	-	-	0.5
	MIC _90_	-	8	32	-	>16	>16	-	-	>16
CPF	MIC/MIC range	>16	0.06–16	>16- >16	8	4- >16	0.5- >16	>16	>16	0.06–2
	MIC _50_	-	0.25	>16	-	>16	>16	-	-	0.5
	MIC _90_	-	2	>16	-	>16	>16	-	-	2
MCF	MIC/MIC range	>16	>16- >16	0.06- >16	>2	>2- >16	0.25- >16	>2	>16	0.008–2
	MIC _50_	-	>16	>16	-	>16	2	-	-	0.12
	MIC _90_	-	>16	>16	-	>16	>16	-	-	>2
AND	MIC/MIC range	>16	>2- >16	0.12- >16	>2	>2- >16	0.25- >16	>2	>16	0.008–1
	MIC _50_	-	>16	>16	-	>16	4	-	-	0.03
	MIC _90_	-	>16	>16	-	>16	>16	-	-	1

Amphotericin B (AMB), Itraconazole (ITC), Voriconazole (VRC), Ravuconazole (RVC), Posaconazole (PSC), Terbinafine (TRB), Caspofungin (CPF) and Micafungin (MCF); Minimum Inhibitory Concentration (MIC), Minimum Effective Concentration (MEC).
